# Increased Levels of 8-Hydroxy-2′-Deoxyguanosine Attributable to Carcinogenic Metal Exposure among Schoolchildren

**DOI:** 10.1289/ehp.7401

**Published:** 2005-05-27

**Authors:** Ruey-Hong Wong, Chung-Yih Kuo, Ming-Lin Hsu, Tsun-Yen Wang, Pi-I Chang, Tsung-Hsun Wu, Shuai Huang

**Affiliations:** Department of Public Health, College of Health Care and Management, Chung Shan Medical University, Taichung, Taiwan

**Keywords:** arsenic, children, chromium, 8-hydroxy-2′-deoxyguanosine, nickel

## Abstract

Arsenic, chromium, and nickel are reported in several epidemiologic studies to be associated with lung cancer. However, the health effects of arsenic, chromium, and nickel exposures are equivocal for children. Therefore, we performed a cross-sectional study to investigate possible associations between the internal concentrations of arsenic, chromium, and nickel and the level of oxidative stress to DNA in children. We measured urinary levels of arsenic, chromium, and nickel for 142 nonsmoking children using atomic absorption spectrometry. As a biomarker for oxidative stress, urinary 8-hydroxy-2′-deoxyguanosine (8-OHdG) levels were analyzed with an enzyme-linked immunosorbent assay kit. The median urinary 8-OHdG level for our subjects was 11.7 ng/mg creatinine. No obvious relationship between the levels of urinary nickel and 8-OHdG was found. Multiple linear regression analysis showed that children with higher urinary chromium had greater urinary 8-OHdG than did those with lower urinary chromium. Similarly, subjects with higher urinary arsenic had greater urinary 8-OHdG than did those with lower urinary arsenic. Furthermore, children with both high urinary arsenic and high urinary chromium had the highest 8-OHdG levels (mean ± SE, 16.0 ± 1.3; vs. low arsenic/low chromium, *p* < 0.01) in urine, followed by those with low arsenic/high chromium (13.7 ± 1.6; vs. low arsenic/low chromium, *p* = 0.25), high arsenic/low chromium (12.9 ± 1.6 vs. low arsenic/low chromium, *p* = 0.52), and low arsenic/low chromium (11.5 ± 1.3); the trend was significant (*p* < 0.001). Thus, environmental carcinogenic metal exposure to chromium and arsenic may play an important role in oxidative DNA damage to children.

Potential hazardous pollutants from industrial sources such as thermal power plants are often emitted to our living environments, where they possibly expose adults and children to heavy metals through inhalation and ingestion of contaminated soil and dust. Earlier literature pointed out that increased use of coal for power production will lead to increased release of metals into the environment (Sabbioni et al. 1984). Furthermore, high quantities of arsenic, chromium, and nickel are detected in milled coal and ash of coal-fired power plants (Goodarzi and Huggins 2001), and these three metals are also reportedly associated with human lung cancers in several occupational epidemiologic studies (Chen and Chen 2002; Droste et al. 1999; Grimsrud et al. 2002). However, the health effects of arsenic, chromium, and nickel exposure are especially equivocal for children. Children are considered to be a population susceptible to adverse health effects induced by air pollutants (Nicolai 1999). Previous studies of metal exposure to children in Taiwan focused primarily on lead in occupational sites (Wang et al. 2002) and Chinese herbal medicine (Cheng et al. 1998), whereas effects of other environmental contaminants such as arsenic, chromium, and nickel on children’s health have been largely ignored.

The carcinogenic potential of arsenic, chromium, and nickel compounds is well established for humans and experimental animals (Hayes 1997). However, the molecular damage formation after exposure to metals is still not well understood. One mechanism proposed frequently is an increase in oxidative DNA lesions attributable to metal exposure, mediated by increased generation of highly reactive oxygen species (ROS) (Dally and Hartwig 1997; Kasprzak 1991; Kasprzak et al. 1997). Oxidative DNA lesions are supposed to play important roles in various diseases including cancer and premature aging (Beckman and Ames 1998; Cerutti 1994; Grisham 1994; Jenner 1994; Witztum 1994). Among the diverse oxidative DNA lesions, 8-hydroxy-2′-deoxyguanosine (8-OHdG) is one of the most abundant base modifications and has attracted special attention because it is premutagenic, causing G-to-T transversions (Cheng et al. 1992); thus, the presence of 8-OHdG may lead to mutagenesis. Moreover, the repair process for 8-OHdG–inflicted damage results in excised 8-OHdG adduct being excreted into the urine (Marnett 2000; Shigenaga et al. 1989). Because of easy collection, urinary 8-OHdG is thus regarded as a suitable biomarker of oxidative stress (Toraason 1999; Wong et al. 2003).

To investigate possible associations between the incorporated internal concentrations of arsenic, chromium, and nickel and the level of oxidative stress in schoolchildren, we performed a cross-sectional study in Taiwan.

## Materials and Methods

### Study areas and subject selection.

The study subjects were nonsmoking fifth-grade pupils (10–12 years of age) who in January 2003 were attending three elementary schools from three different towns of Taichung County, Taiwan. Each selected primary school was close to, and within 1 km of, an ambient air quality monitoring station. Of these schools, Longgang elementary school is also adjacent to the Taichung Thermal Power Plant on the southern side of Taichung harbor, with eight coal-fired generation units in operation to accommodate a total installed capacity of 4,688 MW. The coal consumption of this power plant is approximately 1.28 million tons per year. Coal is stored at and delivered from three coal yards (storage capacity, 310, 0.52, and 0.42 million tons, respectively) near the power plant. Thus, it is possible that coal particles are emitted into the atmosphere from these three coal yards. Additionally, there were no major roads or factories within the Longgang elementary school district. The remaining schools, Shalach and Shuntian, are located in suburban communities and are on the northeastern upwind side approximately 8 and 18 km of the Taichung Thermal Power Plant, respectively. In addition, these two school districts are intersected by major trunk roads.

All subjects who participated in the medical surveillance process underwent physical examinations conducted by a qualified pediatrician. All participating schoolchildren voluntarily entered the study after informed written consent was obtained from the children’s parents. Parents of schoolchildren completed the questionnaire before collecting children’s urine samples. The questionnaire was divided into the following parts: demographic data of the children, respiratory symptoms and diseases of the children, smoking habits and occupation of the parents, and possible sources of indoor air pollution such as household smoking, pet feeding, incense burning all day, and home dampness. According to the responses to the questionnaires, asthma was assessed by the question “has your child had wheezing in the chest accompanied by dyspnea that had ever been given the diagnosis of asthma by a physician during the past 12 months?” Similarly, children who had been diagnosed as having allergic rhinitis by a physician were considered to have a history of allergic rhinitis. Of these study subjects, 16 schoolchildren were excluded from the study because of incomplete urine samples or residence far from the school. Finally, a total of 142 subjects without any disease history except respiratory diseases participated in this study.

### Urine collection.

In our study period, spot morning urine samples were collected in polypropylene specimen containers. The decision to use first morning voids rather than 24-hr collections was based on the report by Thompson et al. (1999), which indicated that 24-hr average urinary levels were not statistically different in values from first voids. Moreover, it is difficult to collect 24-hr urine samples and first voids of morning urine from every subject. Finally, most morning urine specimens we obtained were first spot and small numbers of them were second spot specimens. Immediately after the collection, urine samples were stored at −20°C until used for analysis.

### Measurement of metal levels in urine.

Metal levels in urine samples including arsenic, chromium, and nickel were measured using atomic absorption spectrometry with a graphite furnace (model 4110ZL; Perkin-Elmer, Norwalk, CT, USA) technique and Zeeman background correction. All analytical glassware and plasticware purchased were of low-metal grade and were further cleaned with diluted nitric acid before use. Initially, all the frozen samples were thawed and aliquotted at room temperature. A solution of Triton X-100 (0.1%, wt/vol) was prepared in nitric acid (0.2%, vol/vol). Subsequently, the 18-mL urine samples were diluted with 2 mL of prepared Triton X-100 solution (9:1) and stored at −20°C until required for analysis. Urine test portion and aqueous standards were injected at 20 μL using the autosamplers in the furnace. We used a mixture containing palladium plus magnesium nitrate, and magnesium nitrate as chemical modifiers for the determination of arsenic and chromium in urine, respectively. No matrix modifier was used for the determination of nickel. For analyses of urinary metals, we checked the accuracy of the instrumental methods and the analytical procedure by using reference solutions (standard reference material 12111, normal-range metals urine toxicology control; UTAK Laboratories, Valencia, CA, USA), which were run before every batch of samples. The correlation coefficients for each of the values of the standard curves were all > 0.990. The mean recovery rates ranged from 90 to 105%, and coefficients of variation for reproducibility were all < 10%. Metal concentrations in urine were corrected for each individual according to their urinary creatinine values, and urinary samples were analyzed blind to the status of the individuals for the presence of metals.

### Determination of urinary 8-OHdG levels.

Before examination, urine samples were centrifuged at 2,000 × *g* for 10 min to remove any suspended cell debris. The supernatants were used for the determination of 8-OHdG levels using a competitive enzyme-linked immunosorbent assay kit (ELISA; Japan Institute for the Control of Aging, Fukuroi, Japan). The determination range was 0.5–200 ng/mL. The 8-OHdG monoclonal antibody N45.1 and urine sample were loaded at 50 μL onto a microtiter plate that was coated with 8-OHdG, and incubated at 37°C for 60 min, in accordance with the instructions of the manufacturer. After the wells were washed three times, the antibodies that remained bound to the 8-OHdG in the sample were further bound with the horseradish peroxidase–conjugated secondary antibody, followed by incubation at 37°C for 60 min. The wells were again washed three times. Subsequently, a substrate containing 3,3′,5,5′-tetramethylbenzidine was added, and the wells were incubated at room temperature for 15 min, resulting in the development of color intensity proportional to the amount of antibody bound to the plate. The color reaction was terminated by the addition of stop solution (1 M phosphoric acid), and the absorbance was measured using a computer-controlled spectrophotometric plate reader at a wavelength of 450 nm. The concentration of 8-OHdG in the test samples was interpolated from a standard curve drawn with the assistance of logarithmic transformation. Urinary 8-OHdG levels were subsequently adjusted by urinary creatinine levels.

### Statistical analysis.

Because of the positively skewed distribution of the urinary arsenic, chromium, and nickel levels, we used nonparametric testing to test the differences of urinary metal levels among our study children at three different elementary schools. Similarly, because of the positively skewed distribution of the urinary 8-OHdG levels, we used nonparametric testing to test the differences of urinary 8-OHdG level for each variable. Median values of urinary arsenic, chromium, and nickel levels were used as cutoff. Subsequently, we developed a multiple linear regression analysis to adjust for significant covariate identified in the univariate analysis to evaluate potential differences in urinary 8-OHdG. We also computed regression coefficients and their SEs and calculated least-square means to predict the adjusted 8-OHdG levels for children with different urinary metal contents.

## Results

In total, 142 children (74 boys and 68 girls) were involved in this study. Their ages ranged from 10 to 12 years (mean age, 11.2 years). All study subjects lived within the limits of the Taichung harbor area and lived near their schools. More than half of the parents of study subjects had achieved greater than a senior high school education (58.5% for fathers, 51.4% for mothers). Almost half (49.3%) the parents were smokers. Half the parents were industrial workers. Possible sources of indoor air pollution such as pet feeding were reported in 19.0% of children’s homes, incense burning all day was reported in 40.8% of homes, and home dampness was reported in 20.4% of homes. In addition, prevalences of asthma and allergic rhinitis among study participants were 7.0 and 31.0%, respectively.

The median urinary metal levels were 6.4 μg/L for arsenic, 1.9 μg/L for chromium, and 3.4 μg/L for nickel (Table 1). The creatinine-adjusted median levels were 7.7 μg/g for arsenic, 2.0 μg/g for chromium, and 4.1 μg/g for nickel. Among the 142 urine samples, 19 urine samples were below the detection limit of 0.1 μg/L urine for arsenic, 19 samples were below the detection limit of 0.7 μg/L for chromium, and 16 samples were below the detection limit of 0.8 μg/L for nickel. Especially, the study children at Longgang elementary school had significantly higher urinary levels of arsenic, chromium, and nickel than did those at Shalach and Shuntian elementary schools (*p*-values ≤ 0.01, Kruskal-Wallis test).

Median urinary 8-OHdG level for the study subjects was 11.7 ng/mg creatinine (range, 0.4–59.7 ng/mg creatinine) (Table 2). Children at Longgang elementary school had higher 8-OHdG levels than did those at Shalach and Shuntian elementary schools (p < 0.01, Kruskal-Wallis test). Children of mothers who had greater than a senior high school education had significantly lower 8-OHdG levels than did children of mothers who had less than a senior high school education (p = 0.04, Wilcoxon rank sum test). Children whose parents smoked at home also had significantly higher 8-OHdG levels than did children whose parents did not smoke at home (p = 0.07). Children with allergic rhinitis had significantly lower 8-OHdG levels than did those without allergic rhinitis (p = 0.02). However, sex (p = 0.94), paternal education (p = 0.70), parental occupation (p = 0.11), pet feeding (p = 0.92), incense burning at home all day (p = 0.18), home dampness (p = 0.40), and asthma history (p = 0.51) were not associated with increased urinary 8-OHdG levels.

Children with urinary arsenic levels > 7.7 μg/g creatinine (median, n = 71) had higher 8-OHdG levels than did those with urinary arsenic levels < 7.7 μg/g creatinine (n = 71, p = 0.09). Children with urinary chromium > 2.2 μg/g creatinine (median, n = 71) also had higher 8-OHdG levels than did those with urinary chromium levels < 2.2 μg/g creatinine (n = 71, p = 0.06). However, children with urinary nickel levels > 4.1 μg/g creatinine (median, n = 71) did not have higher 8-OHdG levels than those with urinary nickel levels < 4.1 μg/g creatinine (n = 71, p = 0.62).

Our univariate analysis showed that only elementary school, maternal education, parental smoking status, history of allergic rhinitis, and urinary creatinine-adjusted concentrations of arsenic and chromium were obviously associated with elevated levels of urinary 8-OHdG (p-values < 0.10). Therefore, we performed a multiple linear regression model for urinary 8-OHdG level as a function of maternal education, parental smoking status, history of allergic rhinitis, and urinary creatinine-adjusted concentrations of arsenic and chromium [general linear model (GLM); Table 3]. We excluded elementary school in this model because the variables of elementary school and urinary arsenic and chromium levels had high colinearity. Urinary 8-OHdG level was positively associated with maternal educational level below senior high school (p = 0.05) and was negatively associated with history of allergic rhinitis (p = 0.05). However, parental smoking status (p = 0.47) appeared not to influence the concentrations of urinary 8-OHdG for individuals when examining the data using GLM analysis. Interestingly, a mean difference of 1.9 ng/mg creatinine for urinary 8-OHdG was noted for children with high urinary arsenic levels compared with those with low urinary arsenic levels (p = 0.18). Children with high urinary chromium levels also had a mean difference of 3.0 ng/mg creatinine for urinary 8-OHdG compared with those with low urinary chromium levels (p = 0.04). Furthermore, in this model, the partial R2 value was 17.5% for urinary arsenic and 21.5% for urinary chromium.

**Figure 1:**
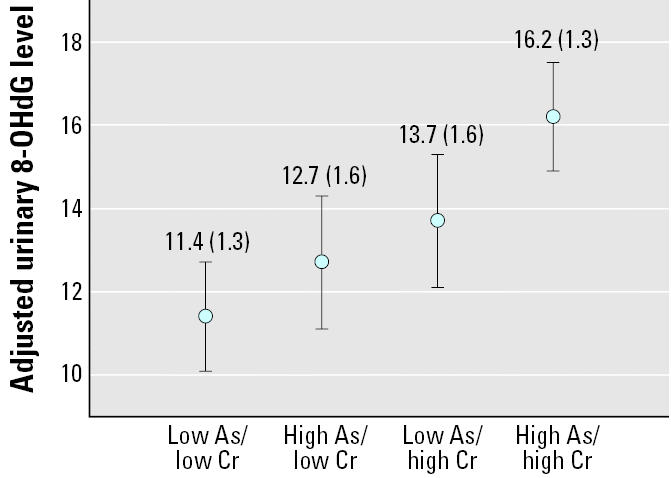
Adjusted urinary 8-OHdG level (ng/mg creatinine) by urinary arsenic and urinary chromium concentrations. Values shown are mean ± SE. Cut points were determined according to medians (arsenic, 7.7 μg/g creatinine; chromium, 2.0 μg/g creatinine) of urinary creatinine-adjusted levels among all subjects.

Subsequently, we performed a least-squares mean analysis to assess the urinary 8-OHdG levels of children with combination of urinary arsenic and chromium adjusted for maternal education and history of allergic rhinitis. Children with both low urinary arsenic and low urinary chromium levels had the lowest urinary 8-OHdG mean levels of 11.4 ng/mg creatinine (n = 44; Figure 1), whereas those with both high urinary arsenic and high urinary chromium levels had the highest urinary 8-OHdG mean levels of 16.2 ng/mg creatinine (n = 43; vs. low arsenic/low chromium, p < 0.01). Those with both low urinary arsenic and high urinary chromium levels (13.7 ng/mg creatinine, n = 28; vs. low arsenic/low chromium, p = 0.25) and those with both high urinary arsenic and low urinary chromium levels (12.7 ng/mg creatinine, n = 27; vs. low arsenic/low chromium, p = 0.52) had a moderately increased 8-OHdG mean levels. This trend in urinary 8-OHdG levels was statistically significant (p = 0.01, GLM). Furthermore, the difference in urinary 8-OHdG levels between the combination high urinary arsenic and chromium and combination low urinary arsenic and chromium (4.8 ng/mg creatinine) was greater than the summation of differences of urinary 8-OHdG levels between the combination low urinary arsenic and high urinary chromium and combination low urinary arsenic and low urinary chromium (2.3 ng/mg creatinine), and combined high urinary arsenic and low urinary chromium with combined low urinary arsenic and low urinary chromium (1.3 ng/mg creatinine).

## Discussion

Attacks on DNA by ROS frequently result in oxidative DNA damage. 8-OHdG is a modified base that occurs in DNA because of attack by hydroxyl radicals. Because it is premutagenic, causing G-to-T transversions (Cheng et al. 1992), the presence of 8-OHdG may lead to mutagenesis. The possibility of 8-OHdG arising from oxidation of deoxyguanosine has been also proposed (Shigenaga et al. 1989), and the result of this deoxyguanosine oxidation does not occur in DNA, so this 8-OHdG has no mutagenic potential. Thus, urinary 8-OHdG is commonly considered a biomarker of oxidative stress, reflecting its repair from DNA. Nonetheless, urinary 8-OHdG has not been used in previous studies to detect the effects of environmental carcinogenic metal exposure in children. In our study, the median value (11.7 ng/mg creatinine) of urinary 8-OHdG for our participants was similar to that of a previous study for normal English children (10.0 ng/mg creatinine) (Drury et al. 1998).

A major fraction of arsenic, chromium, and nickel absorbed by humans appears to be eliminated relatively quickly and mainly via urine. The biologic half-life for these metals has been estimated to be between 1 and 3 days (Hwang et al. 1997; Paustenbach et al. 1997; Sunderman 1993; Vahter 1994). Thus, these metal concentrations in urine samples are determined as important short-term exposure biomarkers and have been used in many epidemiologic studies (Moore et al. 1997; Smith-Sivertsen et al. 1998; Stern et al. 1998). In the present study, we found statistically significant relationships between the urinary concentrations of chromium and arsenic and the level of DNA oxidative stress. The lack of correlation between exposure to nickel and DNA oxidative stress could be attributable to a low biologically relevant dose in the study population. Several studies have demonstrated that arsenic and chromium cause oxidative DNA damage to cultured cells (Kessel et al. 2002; Yuann et al. 1999). Previous epidemiologic studies have also shown increased 8-OHdG levels in humans exposed to arsenic (Matsui et al. 1999) or chromium (Kuo et al. 2003). Transition metals are commonly thought to produce ROS such as hydroxyl radicals that can directly damage cellular DNA. The other mechanism is indirect oxidative DNA damage due to inflammation caused by metal exposure (Donaldson et al. 2002). Some metals may stimulate the defense systems of the body so that they react against the toxic damage to produce cytokines (Carter et al. 1997; Donaldson et al. 2002). Several cytokines cause production of large amounts of ROS. Some propose that ROS generated in inflamed tissues can cause injury to target cells and also damage DNA, which contributes to carcinogenic processes (Chazotte-Aubert et al. 1999; Eiserich et al. 1998).

Environmentally relevant metals seldom occur alone. Little is known about the exact mechanism of carcinogenesis of two or more metals when they are present together. It is generally assumed that the concept of additivity is operative on low-level exposures to chemical mixtures (Hartwig and Schwerdtle 2002). It is particularly interesting that we observed a synergistic effect for combined arsenic and chromium exposure on DNA oxidative stress in the present study (Figure 1). It may be that other types of cellular damage are caused by metal exposure, which also contributes to their carcinogenic potentials. There is accumulating evidence that metals including arsenic and chromium can interfere with distinct steps of diverse DNA repair systems (Hartwig and Schwerdtle 2002). Thus, oxidative DNA lesions are not only induced by metals at biologically relevant concentrations, but their extent may also be enhanced indirectly by impaired repair. Further studies are required to clarify these findings.

The amount of the modified base in cellular DNA excreted into urine should represent the average rate of DNA damage in the whole body (Cooke et al. 2000). Thus, it is possible that the levels of oxidative DNA damage are reflective of different active diseases, especially active inflammation (Wong et al. 2003). Besides, urinary levels of any oxidative lesion rely on efficient renal excretion of the damage products, so renal impairment can therefore affect urinary 8-OHdG levels (Akagi et al. 2003). In our study, urinary creatinine levels were used to correct for variation in urine concentration. In addition, no medical histories were reported by our participants except asthma and allergic rhinitis. There is ample evidence indicating that allergic disorders, such as asthma and rhinitis, are mediated by oxidative stress (Bowler and Crapo 2002). In our study, we did not observe a significant association between asthma and urinary 8-OHdG level in children. On the contrary, children with allergic rhinitis had significantly lower 8-OHdG than did those without. One could interpret this finding as an effect rather than a cause; that is, children with past allergies or past episodes of respiratory symptoms have had previous medical care, and their parents may have been urged to improve their environment to alleviate the symptoms. We also found that maternal education level, used as a proxy for socioeconomic status, was significantly related to children’s oxidative DNA damage. Maternal education may convey information that influences the patterns of potential metal exposure as well as health care for children. In addition, we also observed that children whose parents smoked had higher 8-OHdG expression, although it was not significant in our multiple linear regression model. Because cigarette smoke contains ROS, the association between cigarette smoking and urinary 8-OHdG has been previously reported (Loft and Poulsen 1996). However, the association between 8-OHdG and children exposed to environmental tobacco smoke has not been investigated. Oxidative damage occurs rapidly after exposure, and this damage can be repaired rapidly. Because Taiwanese children may not have regular chances to be exposed to tobacco smoke from their family members, the effects of passive smoking on 8-OHdG in children are less likely to appear in the model.

Children spend their most of their time indoors. It is therefore important to consider the effects that exposure to indoor air pollutants may have on children’s oxidative DNA damage. House dust and fungi are the major indoor pollutants in our subtropical area (Li et al. 1994; Wang et al. 1999). In addition, burning Chinese incense releases polycyclic aromatic hydrocarbons (Lung et al. 2003), which may increase cellular oxidative stress (Wu et al. 2003). Our data provide no evidence for an association between the levels of indoor environmental factors such as pet feeding, incense burning, and home dampness. In our study, these indicators were self-reported and therefore were subjective and could have resulted in misclassification of exposure that might reduce the observed associations.

When the balance between pro-oxidant and antioxidant processes is shifted in favor of the former, increased 8-OHdG would be generated from further DNA oxidation and ring opening followed by rearrangements (Cadet et al. 2003). However, the role of pro-oxidant and antioxidant on 8-OHdG in our study children was not identified. In addition, the 8-OHdG monoclonal antibody used in our ELISA assay has similar binding affinity for the oxidized free base 8-hydroxyguanine, and the oxidized nucleoside 8-hydroxyguanosine (Yin et al. 1995). Thus, the possibility of overestimation of urinary 8-OHdG levels by our ELISA assay cannot be ruled out, and this bias would be likely to attenuate the observed association if it was nondifferential.

In our study, children’s exposure to arsenic and chromium was associated with increased generation of subsequent 8-OHdG. However, the role of other carcinogenic metals on oxidative DNA damage also requires further study. For the future, a longitudinal rather than a cross-sectional study should be conducted to ascertain the possible association between carcinogenic metal exposure and oxidative DNA lesions. A longitudinal study that includes a relevant number of environmentally exposed participants offers an advantage for studying dose–effect relationships over time with repeated measurements.

## Figures and Tables

**Table 1:** Concentrations of arsenic, chromium, and nickel in the study population.

	Elementary school			All (*n* = 142)			
Variable	Longgang (*n* = 49)	Shalach (*n* = 45)	Shuntian (*n* = 48)	Mean ± SE	Geometric mean	Median	Maximum
Arsenic (μg/L urine)	16.2 ± 2.6**	8.7 ± 0.9	3.7 ± 0.6	9.1 ± 1.1	3.9	6.4	86.4
Arsenic (μg/g urinary creatinine)	21.3 ± 4.3**	10.0 ± 1.2	4.7 ± 0.7	12.1 ± 1.6	5.0	7.7	149.3
Chromium (μg/L urine)	2.6 ± 0.2**	1.9 ± 0.1	1.3 ± 0.1	1.9 ± 0.1	1.7	1.9	6.6
Chromium (μg/g urinary creatinine)	3.7 ± 0.5**	2.2 ± 0.2	2.0 ± 0.2	2.7 ± 0.2	2.2	2.0	21.4
Nickel (μg/L urine)	5.4 ± 0.5*	3.9 ± 0.3	2.8 ± 0.2	4.0 ± 0.2	3.2	3.4	14.5
Nickel (μg/g urinary creatinine)	7.2 ± 1.1**	4.6 ± 0.5	4.1 ± 0.5	5.4 ± 0.4	4.0	4.1	47.8

All values shown are mean ± SE.

* p = 0.01

** p < 0.01, Kruskal-Wallis test.

**Table 2:** Urinary 8-OHdG (ng/mg) creatinine stratified by different variables.

Variable	No.	Mean ± SE	Median (range)
Total	142	13.6 ± 0.7	11.7 (0.4–59.7)
Elementary school			
Longgang	49	18.8 ± 1.5	18.8 (3.9–59.7)**
Shalach	45	9.8 ± 0.7	9.9 (0.4–22.1)
Shuntian	48	11.8 ± 0.9	11.6 (0.7–37.3)
Sex			
Boys	74	13.2 ± 0.9	12.0 (0.4–39.7)
Girls	68	14.0 ± 1.1	11.3 (2.5–59.7)
Paternal education (years)			
> senior high school (≥ 12)	83	12.8 ± 0.7	11.4 (2.5–33.0)
< senior high school (< 12)	59	14.7 ± 1.4	12.0 (0.4–59.7)
Maternal education (years)			
> senior high school (≥ 12)	73	11.8 ± 0.7	10.8 (0.8–31.3)*
< senior high school (< 12)	69	15.5 ± 1.2	13.0 (0.4–59.7)
Parental occupation			
Industry	72	14.8 ± 1.1	12.8 (0.7–59.7)
Nonindustry	70	12.4 ± 0.9	10.6 (0.4–33.0)
Parental smoking status			
Yes	70	14.3 ± 0.9	12.8 (0.7–37.3)#
No	72	12.9 ± 1.1	10.6 (0.4–59.7)
Possible indoor pollutants			
Pet feeding			
Yes	27	13.3 ± 1.6	11.1 (3.2–33.1)
No	115	13.7 ± 0.8	12.0 (0.4–59.7)
Incense burning at home all day			
Yes	58	14.5 ± 1.1	11.1 (0.4–59.7)
No	84	12.9 ± 0.9	13.5 (0.8–39.7)
Home dampness			
Yes	29	15.3 ± 2.1	12.0 (2.5–59.7)
No	113	13.1 ± 0.7	11.4 (0.4–39.7)
Personal medical histories			
Asthma			
Yes	10	12.2 ± 2.3	10.1 (5.2–25.1)
No	132	13.7 ± 0.8	11.9 (0.4–59.7)
Allergic rhinitis			
Yes	44	11.0 ± 0.9	9.9 (0.4–25.1)*
No	98	14.8 ± 0.9	12.3 (0.7–59.7)
Adjusted urinary arsenic^a^			
High (≥ 7.7 μg/g creatinine)	71	14.8 ± 1.1	12.4 (0.4–59.7)#
Low (< 7.7 μg/g creatinine)	71	12.4 ± 0.9	11.1 (0.7–39.7)
Adjusted urinary chromium^a^			
High (≥ 2.0 μg/g creatinine)	71	15.5 ± 1.3	12.4 (0.4–59.7)#
Low (< 2.0 μg/g creatinine)	71	11.6 ± 0.6	11.1 (3.1–24.5)
Adjusted urinary nickel^a^			
High (≥ 4.1 μg/g creatinine)	71	13.8 ± 1.2	11.4 (0.4–59.7)
Low (< 4.1 μg/g creatinine)	71	13.3 ± 0.8	12.3 (0.7–39.7)

a Cut points were determined according to medians of urinary creatinine-adjusted levels among all subjects.

* 0.01 < p < 0.05;

** p < 0.01;

# 0.05 < p < 0.10.

**Table 3:** Multiple linear regression model between the level of urinary 8-OHdG and incorporated concentration of arsenic and chromium (n = 142).

Component model	Model parameter (SE)	Significance (p-value)	Explained variance (%)
Maternal education (< senior high school vs. > senior high school)	2.8 (1.4)	0.05	34.7
Parental smoking status (yes vs. no)	1.0 (1.4)	0.47	2.2
Allergic rhinitis (yes vs. no)	−3.0 (1.6)	0.05	24.1
Adjusted urinary arsenic (high vs. low)^a^	1.9 (1.4)	0.18	17.5
Adjusted urinary chromium (high vs. low)^a^	3.0 (1.4)	0.04	21.5

a Cut points were determined according to medians (arsenic, 7.7 μg/g creatinine; chromium, 2.0 μg/g creatinine) of urinary creatinine-adjusted levels among all subjects.
